# [^177^Lu]Lu-DOTA-TATE tumour and organ time-activity curves: prediction from a single-time-point [^68^Ga]Ga-DOTA-TATE PET/CT measurement

**DOI:** 10.1186/s40658-025-00826-4

**Published:** 2026-01-16

**Authors:** Valentina Vasić, Johan Gustafsson, Elham Yousefzadeh-Nowshahr, Ambros J. Beer, Katarina Sjögreen Gleisner, Gerhard Glatting

**Affiliations:** 1 Department of Nuclear Medicine, Ulm University, 89081 Ulm, Germany; 2 Medical Radiation Physics, Department of Nuclear Medicine, Ulm University, Ulm, Germany; 3 Medical Radiation Physics, Lund University, Lund, Sweden

**Keywords:** PRRT, PBPK model, Single-Time-Point [^68^Ga]Ga-DOTA-TATE, Error model analysis, TAC prediction

## Abstract

**Aim:**

Predicting the time-activity curve (TAC) of [^177^Lu]Lu-DOTA-TATE for organs at risk and neuroendocrine tumours (NETs) is an essential element in the calculation of the absorbed dose (AD) and a critical step for individualising peptide receptor radionuclide therapy (PRRT) treatment planning. This study aims to predict the TAC of [^177^Lu]Lu-DOTA-TATE using a single quantitative image of [^68^Ga]Ga-DOTA-TATE and population data with a physiologically based pharmacokinetic (PBPK) model.

**Methods:**

A PBPK model was developed for [^68^Ga]Ga-DOTA-TATE and [^177^Lu]Lu-DOTA-TATE, including organs and NETs. To generate reference TACs, general physiological parameters were taken from the literature, while individual model parameters were estimated using pre-therapy (PET/CT) and post-therapy (planar and SPECT/CT) image-based organ activity measurements from patients with NETs. Different error models were evaluated to determine the best one. To predict the TAC of [^177^Lu]Lu-DOTA-TATE from a single [^68^Ga]Ga-DOTA-TATE PET/CT, individual model parameters were estimated using only [^68^Ga]Ga-DOTA-TATE organ and tumour activity measurements. Finally, the predicted [^177^Lu]Lu-DOTA-TATE TACs for modelled organs and NETs were compared to the reference.

**Results:**

The best error model was the proportional data-based error model, where the proportionality parameter *b* differs between diagnostic and therapeutic data, and between tumours and organs: b_T, Organ_, b_T, Tumour_, and b_D, Organ_, b_D, Tumour_. The medians for b_T, Organ_, b_T, Tumour_ and b_D, Organ_, b_D, Tumour_ were determined to be 0.16, 0.39, 0.35, and 0.27, respectively. For the prediction, b_D, Organ_ and b_D, Tumour_ were used as patient-specific proportional errors. The relative prediction error (RPE) was calculated for the predicted time-integrated activity (TIA). The mean and standard deviation for the RPEs were found to be (− 5 ± 51)%, (− 4 ± 22)%, (− 13 ± 40)%, and (− 10 ± 21)% for tumours, kidneys, liver, and spleen, respectively. The mean absolute percentage errors (MAPEs) were 43%, 18%, 31% and 17% for tumours, kidney, liver, and spleen, respectively.

**Conclusion:**

The integration of the PBPK model with a data-based proportional error model represents a significant improvement in predicting TACs for estimating tumour and organ ADs following [^177^Lu]Lu-DOTA-TATE therapy, using single-time-point PET/CT imaging with [^68^Ga]Ga-DOTA-TATE. These results emphasise the importance of error model analysis in PBPK modelling.

**Supplementary Information:**

The online version contains supplementary material available at 10.1186/s40658-025-00826-4.

## Introduction

Peptide receptor radionuclide therapy (PRRT) with radiolabelled somatostatin analogues (SSAs) is an efficacious and well-tolerated treatment for neuroendocrine tumours (NETs) [1]. The approved PRRT with [^177^Lu]Lu-DOTA-TATE involves administering a fixed activity of 7.4 GBq/cycle for four cycles with 8-week intervals between cycles [2]. In recent years, numerous research groups have investigated the prediction of therapy absorbed doses (ADs) to individualise treatments for patients [3–14]. AD estimation requires knowledge of the time-integrated activity (TIA) and the dose-conversion factor [15]. Among these, the TIA shows considerably higher inter-patient variability [16], underscoring the importance of its precise determination for reliable dosimetry [17]. Consequently, individualised biokinetic modelling of time–activity curves (TACs) is fundamental for obtaining robust estimates of the TIA coefficient. Physiologically based pharmacokinetic (PBPK) models offer a means to integrate fundamental pharmacokinetic principles in this modelling, as well as to generalise from previously collected patient data. Such advanced modelling may thus be used to support treatment planning and optimisation [18, 19]. Among these, PBPK models incorporate a greater degree of physiological prior knowledge, enabling more accurate dosimetry. Moreover, PBPK models offer substantial advantages in representing biological systems and providing predictive insights, thereby supporting treatment optimisation and contributing to improved therapeutic outcomes [9, 20]. The selection of patients suitable for PRRT is guided by SSA-based PET imaging, which evaluates tumour burden and expression of somatostatin receptors (SSTR) in individual lesions [21, 22]. Pre-therapy molecular imaging could help tailor [^177^Lu]Lu-DOTA-TATE therapy by optimising ADs to target tumour lesions while ensuring organ-at-risk thresholds (such as kidneys and red marrow) are met. While the impact of this approach may be most relevant in guiding the first therapy cycle, it supports a personalised treatment strategy from the outset.

As suggested by Hardiansyah et al. [6], kinetic modelling has the potential to improve the extrapolation of early imaging time points for [^68^Ga]Ga-DOTA-TATE, enabling the prediction of ADs of [^177^Lu]Lu-DOTA-TATE to tumours and organs. Moreover, the findings of Hänscheid et al. [3] suggest that PET with [^68^Ga]Ga-labelled SSAs could be used to assess tumour radionuclide uptake before PRRT in meningiomas and to estimate the AD expected for therapy. Also, Standardised Uptake Value (SUV) evaluation obtained from pretherapeutic [^68^Ga]Ga-DOTATOC PET can predict the ADs to NETs during PRRT, aiding in selecting the most suitable candidates for this therapy [4]. These results suggest that pretherapy ^68^Ga-labelled SSAs PET carries valuable information on ADs of PRRT. Some studies have investigated the prediction of therapeutic ADs based on pretherapeutic PET imaging [5–9, 13, 21]. However, for [^177^Lu]Lu-DOTA-TATE therapy, the results showed a difference of about 50% between predicted and actual ADs, emphasising the need for improvement [21]. Moreover, no analysis of error models has been conducted to date to improve the fitting of TACs to measured data, i.e., how uncertainties in measured data are considered within the modelling. An assumption of poor measurement precision (large uncertainties) could yield therapy predictions that do not sufficiently account for measured data, whereas an assumption of good precision (low uncertainties) would lead to interpreting measurement errors as a real signal. Consideration of measurement uncertainties is thus important in modelling and time-activity curve fitting.

Since ADs are directly dependent on TIAs [23, 24], knowing the TACs is critical for accurate prediction of ADs. Biokinetics can be measured at fixed times during therapy. However, predicting the TAC solely from diagnostic information is not straightforward due to various factors, including chemical differences between pharmaceuticals, variations in administration procedures, and biological and technical uncertainties, such as image noise. Knowledge of population information integrated into a PBPK model, together with an individual [^68^Ga]Ga-DOTA-TATE PET/CT measurement, could be sufficient for accurate and precise prediction of [^177^Lu]Lu-DOTA-TATE TACs and, consequently, the ADs delivered to organs at risk and NETs during therapy [9, 21].

This study aims to evaluate the potential of using a PBPK model with a single [^68^Ga]Ga-DOTA-TATE PET/CT uptake measurement and Bayesian information to predict TACs and, consequently, TIAs of tumours and organs after [^177^Lu]Lu-DOTA-TATE administration, including error analysis on measured diagnostic and therapeutic data.

## Methods

### Patient data

Twelve patients (8 males, 4 females, ($65\pm12$) years) with metastatic NETs received a pre-therapeutic [^68^Ga]Ga-DOTA-TATE injection followed by [^177^Lu]Lu-DOTA-TATE therapy (Table 1). The patient data originated from the Iluminet trial [25], for which dosimetry has been published elsewhere [10]. Patients received an injection of $(14.9\pm7.0)$ nmol of peptide with ($0.20\pm0.05$) GBq of [^68^Ga]Ga-DOTA-TATE for PET/CT imaging. For therapy, patients received ($134\pm10$) nmol of the peptide with ($7.46\pm0.07$) GBq of [^177^Lu]Lu-DOTA-TATE at Skåne University Hospital, Lund [10].

**Table 1:** Patient characteristics

Characteristic	Median [min, max]
Sex	
Male	8
Female	4
Age [years]	67 [35, 80]
Weight [kg]	76.5 [56, 124]
Height [cm]	176.5 [157, 193]
Glomerular filtration rate (GFR) [mL/min/1.73 m^2^]	0.07 [0.06, 0.11]
Applied activity [GBq]	7.47 [7.32, 7.54]
Volume [L]	
Kidneys	0.36 [0.20, 0.41]
Spleen	0.18 [0.07, 0.35]
Liver	1.80 [1.25, 3.31]
Tumour	0.03 [0.01, 0.06]
Total tumour in the body	0.11 [0.03, 0.29]
Therapeutic percentage of tumour in the liver [%]	8.24 [0.99, 35.73]
Diagnostic percentage of tumour in the liver [%]	2.44 [1 × 10^− 6^, 15.02]
Infusion unlabelled therapy [nmol]	123 [104, 141]
Infusion radiolabelled therapy [nmol]	10.28 [10.07, 10.37]
Injection unlabelled diagnostic [nmol]	13 [9, 36]
Injection radiolabelled diagnostic [nmol]	1.7 [1.1, 2.8] × 10^− 3^
Infusion duration [min]	36 [34, 38]

#### [^68^Ga]Ga-DOTA-TATE PET/CT imaging

PET/CT imaging was performed at a median of 5.1 weeks (range 0.3 to 18 weeks) before the first treatment cycle using a GE Discovery PET/CT 690 system (GE HealthCare). The time interval between tracer administration and imaging was (63 ± 5) min, with each bed position imaged for 3 min. Further details on image acquisition, reconstruction, segmentation, partial-volume correction, and activity quantification were provided by Stenvall et al. [10]. Table 1 reports the median, minimum, and maximum of organ and tumour volumes.

#### [^177^Lu]Lu-DOTA-TATE Peri-therapeutic imaging

SPECT/CT images were obtained at (22 ± 1) h post-injection, using a GE Discovery 670 system with medium-energy collimators and energy windows centred at 208 keV with a 15% width [10]. Whole-body planar images were acquired at four time points: 1 h, day 1, day 4, and day 7 after therapeutic administration. The planar images were acquired using the same camera systems as for SPECT/CT imaging [10].

### PBPK model

A [^68^Ga]Ga-DOTA-TATE and [^177^Lu]Lu-DOTA-TATE whole-body PBPK model previously created and validated [26, 27] was developed and implemented in SimBiology (MATLAB^®^) version 2021a. The model comprises seven organs that express somatostatin-receptor 2 (SSTR2): gastrointestinal tract (GI), liver, spleen, muscles, red bone marrow (RM), prostate, adrenal gland, and the rest of the body (RoB). It also contains six non-specific SSTR2-binding organs (heart, bone, fat, lung, skin, brain). Organs exhibiting non-specific binding typically comprised a vascular compartment alongside an interstitial compartment. In contrast, organs demonstrating specific binding included three additional compartments: receptor binding, endosomal, and lysosomal space [27].

The kidney model includes additional compartments for the proximal tubule, collecting ducts, and nonspecific accumulation [28], distinguishing it from organs with specific binding [27].

Tumours are represented as SSTR2-receptor-expressing organs. The two largest NETs (volume > 5 mL) (Tu1 and Tu2), clearly identifiable in planar images and that did not overlap with other tissues or organs with a high activity accumulation, were selected for the analysis. Other tumour lesions were liver tumours (TuL) and tumours in the rest of the body (TuRofB), which represent tumours that were not Tu1, Tu2, or TuL. The TuL and TuRofB fit parameters (receptor density, flow, and sorting) were set to the average values of the parameters found for Tu1 and Tu2. All organs are connected by blood flow through veins and arteries [27] (Supplements, section S1).

The patient parameter values were taken from the whole-body PBPK models published in previous studies for [^177^Lu]Lu-PSMA [29, 30], alpha particle generator models [28, 31] or [^90^Y]Y-DOTA-TATE [26] and for [^177^Lu]Lu-DOTA-TATE [27]. Additional model parameter values were taken from the literature, including permeability surface area [32], serum flows for organs [33, 34], masses of unmeasured organs [35], dissociation constants [36], or from direct measurements on the patient, such as height, body weight, glomerular filtration rate (GFR), and organ volumes. All parameters are detailed in the Supplements [27].

**Fig. 1:**
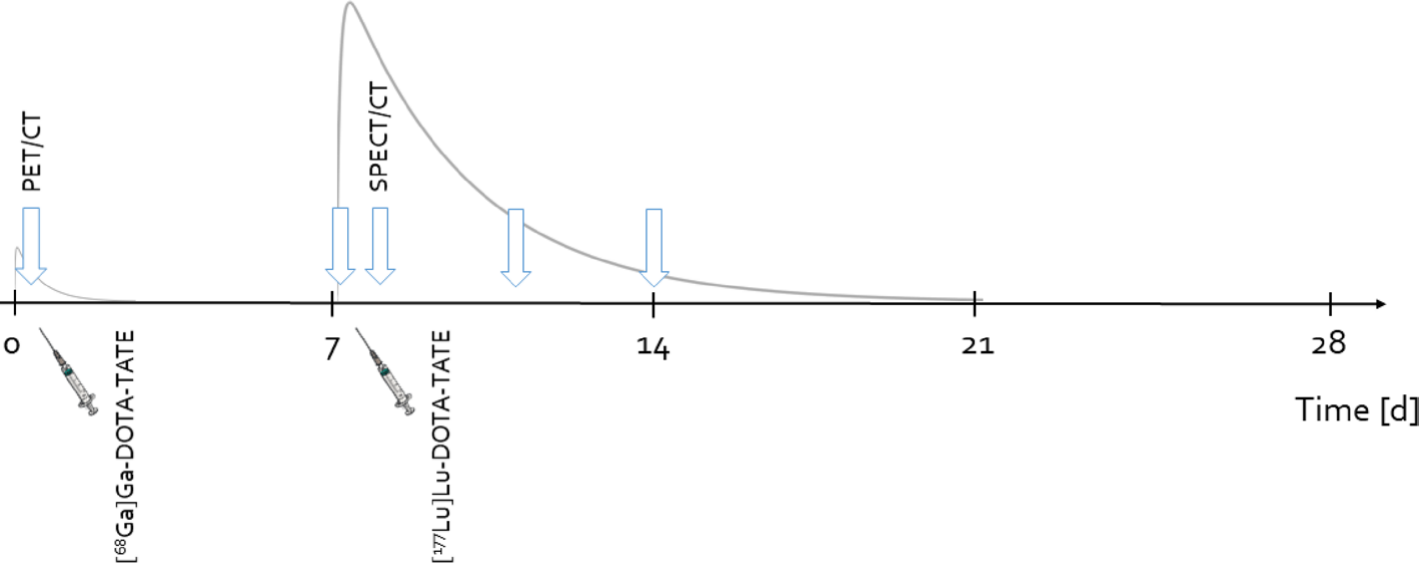
Timeline illustrating the measured data incorporated into the PBPK model. At time 0 d, a [^68^Ga]Ga-DOTA-TATE injection is administered, followed by a PET/CT scan 1 h after injection. On day 7, the [^177^Lu]Lu-DOTA-TATE infusion is administered, followed by planar (1 h, 1 d, 4 d, 7 d) and SPECT/CT (1 d) measurements. The simulation stops on day 28

The PBPK model used 10 adjustable parameters. These parameters were the receptor densities of the spleen, liver, kidney, and muscle with fat, the receptor densities of Tu1 and Tu2, the serum flows of the two tumours, and the sorting and degradation parameters. A single sorting and a single degradation parameter were assumed for all organs; this was based on the observation in Vasić et al. [27] that these parameters were equal between organs when considering the uncertainties of the fitted parameters (Table S3 in the supplementary data of Vasić et al. [27]). [^68^Ga]Ga- and [^177^Lu]Lu-DOTA-TATE pharmacokinetics were simulated by the PBPK model. The injection and infusion were implemented according to the timeline illustrated in Fig. 1. The details of the simulation and fitting method were reported in the Supplement of Vasić et al. [27].

### Measurement error model selection and parameter estimation

The following algorithm was used to determine the error model for the data.

First, for *i* measured time-activity data, the model-based combined error model was investigated [19]:

1 $${\sigma}_{i}=a+b \cdot {f(t}_{i}\left|\boldsymbol{p}\right),$$

where *a* and *b* are two parameters describing the constant and proportional component of the uncertainty; ${f(t}_{i}\left|\boldsymbol{p}\right)$is the model value at time point ${t}_{i}$ that depends on the model parameters $\boldsymbol{p}$. The error model parameters *a* and *b* were estimated by fitting the model to the measured time-activity data and determined individually for each patient. The medians of the individually fitted values of the 12 patients were then assumed to provide a reasonable estimate for the actual values of *a* and *b*.

Since the fitted median *a* was small, i.e. $a < 10^{{ - 6}} < < {\text{ }}b \cdot f(t_{i} ,\vec{p})$, *a* was set to zero subsequently. Thus, the proportional error model was used further.

The value of parameter b was determined analytically from the objective function (OF) by solving $\frac{{\partial OF}}{{\partial b}} = 0$ [37] yielding

2 $${b}_{model-based}=\sqrt{\frac{1}{N}\sum_{i=1}^{N}\frac{{({y}_{i}-{f(t}_{i}|\boldsymbol{p}\left)\right)}^{2}}{{{f(t}_{i}\left|\boldsymbol{p}\right)}^{2}}}$$

where ${y}_{i}$ are the measured time-activity data and *N* the number of measurements for the investigated patient.

Thus, the variance of the model-based proportional error model was obtained using Eq. 3:

3 $${\sigma}_{model-based}^{2}={({b}_{model-based} \cdot {f(t}_{i}|\boldsymbol{p}\left)\right)}^{2}$$

The data-based proportional error model [38] was determined analogously to be

4 $${b}_{data-based}=\sqrt{\frac{1}{N}\sum_{i=1}^{N}\frac{{({y}_{i}-{f(t}_{i}|\boldsymbol{p}\left)\right)}^{2}}{{{y}_{i}}^{2}}}$$

The variance of the data-based error model was

5 $${\sigma}_{data-based}^{2}={({b}_{data-based} \cdot {y}_{i})}^{2}$$

Three methods for determining the proportional error parameter, *b*, were investigated.

**Fig. 2:**
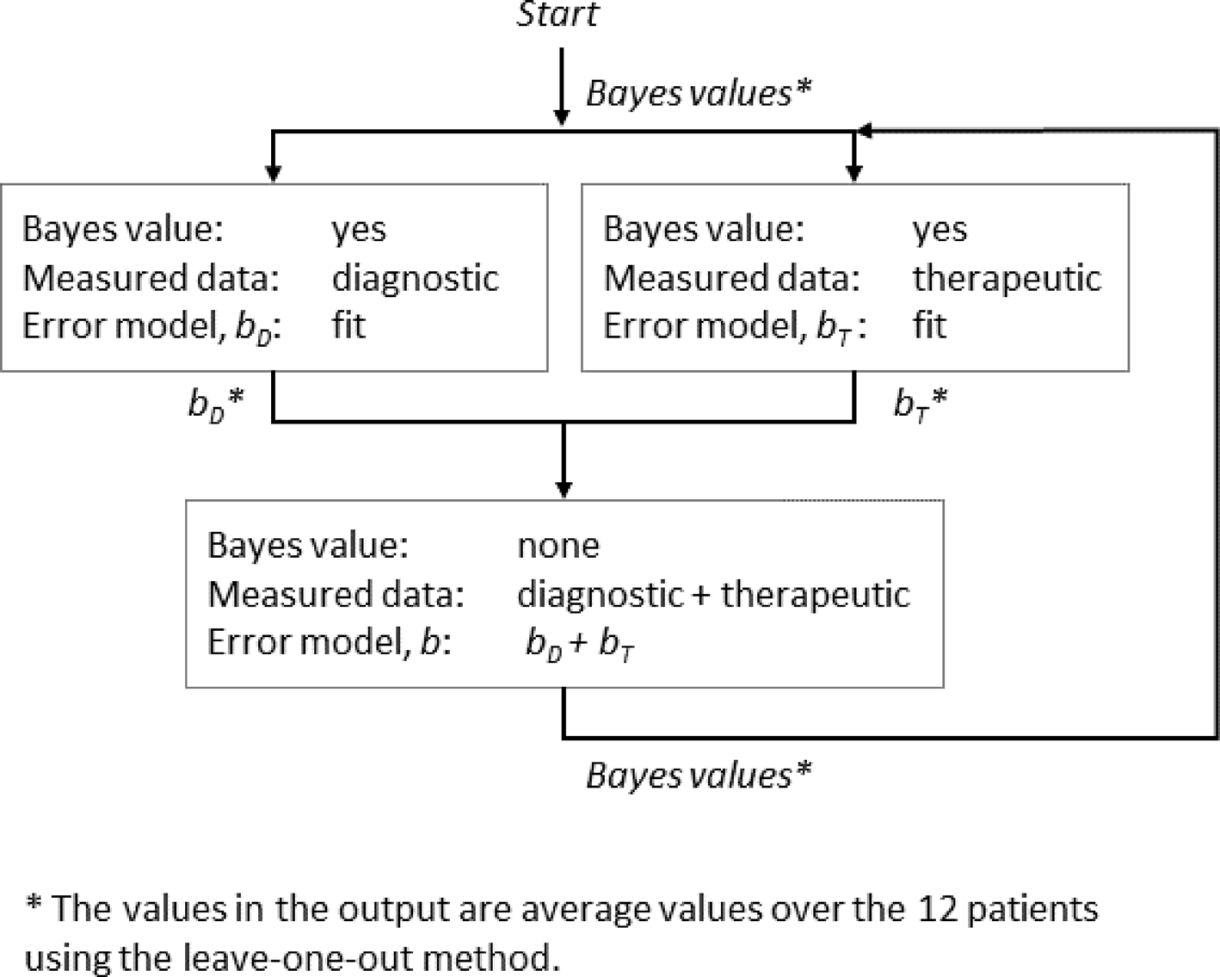
Workflow for estimating the parameters ${b}_{D}$ and ${b}_{T}$ of the proportional error model. Iterations were performed from the first to the second block until the proportional error converged, i.e., 28 iterations

Diagnostic measurements were performed with a PET/CT system, whereas therapeutic measurements were acquired with a SPECT/CT system combined with planar acquisitions. Since the data acquisition methods differ, a different proportionality constant *b* was assumed for each modality. Therefore, in **Method I** (Fig. 2), diagnostic and therapeutic measured time-activity data were separated, i.e., the first fitting procedure exclusively incorporated PET/CT measurements, and from this, the proportional error parameter ${b}_{D}$ was estimated according to Eq. 2; the second fitting procedure incorporated therapeutic measurements (SPECT/CT and planar measurements), where the proportional error ${b}_{T}$ was also estimated according to Eq. 2. The third fitting was done to estimate Bayes values that were required for the first and second fitting in the next iteration. This third fitting was done using all data (diagnostic and therapeutic), using the median ${b}_{D}$ and ${b}_{T}$ calculated in the corresponding iteration. The calculation was iterated 28 times to ensure convergence of both *b* parameters and the Bayesian values for the model fit parameter estimations (Fig. 2).

In the analysed patients, the organs exhibited larger volumes than those of the tumours. Moreover, the biokinetics of organs and tumours differed substantially [39, 40]. Therefore, it was hypothesised that the proportional parameter *b* might differ in the estimation of the TAC. To investigate this, in **Method II**, different errors for organs and tumours were assumed. In addition to the initial set of 10 fit parameters also ${b}_{D,Organ}$, ${b}_{D,Tumour}$, ${b}_{T,Organ}$ and ${b}_{T,Tumour}$ were fitted according the workflow in Fig. 2. For the value of ${b}_{D,Organ}$, ${b}_{D,Tumour}$, ${b}_{T,Organ}$ and ${b}_{T,Tumour}$ 5, 3, 24 and 9 time-activity data were associated in the fit, respectively.

In the fitting procedures implemented in MATLAB, the built-in model-based data error approach was used. However, since the aim was to make predictions, only the measured data were available, and the actual uncertainty of the measured data could, in principle, be determined, allowing the estimation of the proportional constant *b*. Therefore, our analysis was extended to the data-based error model. In **Method III**, the procedure followed was identical to that of Method II; however, instead of using the model-based proportional error model (Eqs. 2 and 3), the data-based error model (Eqs. 4 and 5.) was applied. Under the hypothesis that the proportional constant *b* can be experimentally characterised for PET data, this information can be considered patient-specific. Consequently, the prediction of **Method III** focused on the implementation of the proportional constant ${b}_{D,Organ}$ and ${b}_{D,Tumour}$ as patient specific data, and ${b}_{T,Organ}$ and ${b}_{T,Tumour}$ taken as median of the population.

### Prediction of therapeutic TACs

To predict the TAC of [^177^Lu]Lu-DOTA-TATE from the single-time-point [^68^Ga]Ga-DOTA-TATE PET/CT, population information was used for the 10 fit parameters, as Bayes parameter values. This is essential because the number of measured data (8 data points) is fewer than the fit parameters [10].

#### Bayesian information

Bayes parameter values of the 10-fit-parameter model were estimated by collecting the fitted values from the 12 patients. Mean and standard errors were calculated using the leave-one-out method, i.e., a cross-validation technique. Given the data from 12 patients, one observation (i.e., the dataset of one patient) was removed, and the mean and standard deviation of the observation were calculated using the data of the remaining 11 patients. These mean and standard deviation values served as the Bayesian parameters for the left-out patient of the dataset. The procedure was repeated for each patient: for the second patient, the Bayesian mean and standard deviation were calculated based on the data from the remaining 11 patients, and so forth. In this way, the patient under study did not contribute information about themselves to their own Bayesian values.

The Bayes parameter values were the patient averages and standard deviations of the last iteration of the case studied in the error model analysis.

#### Reference TACs

To estimate the TACs for the reference method, all measured data were used, i.e. the time-activity data from the diagnostic PET/CT measurements and the peri-therapeutic planar and SPECT/CT measurements. Three reference methods were evaluated: first, the model-based error model with two *b* parameters (Method I), second, the model-based error model with four *b* parameters (Method II) and third, using the data-based error model with four *b* parameters (Method III).

Bayesian information, obtained from the last iteration of analysis conducted on the error model and reported in Fig. 2, was also included in the fit, and initial values for the fitting parameters were used from Vasić et al. [27].

The goodness of fit was evaluated by visual inspection and according to the criteria reported in Kletting et al. [18], i.e., the coefficient of determination was close to zero; the coefficient of variation (CV) was acceptable (CV < 50% [41]); values of the correlation matrix were in an acceptable range as proposed by Wastney et al. (− 0.8 < each element < 0.8) [41]; and Akaike information criterion was compared with values of other functions from Vasić et al. [27].

#### Individual [^177^Lu]Lu-DOTA-TATE TAC prediction

To predict the TACs of [^177^Lu]Lu-DOTA-TATE, the 10 unknown patient-specific model parameters were fitted using individual [^68^Ga]Ga-DOTA-TATE PET/CT and the leave-one-out Bayesian information only. Three different predictions were calculated, using the three proportional error models: Methods I, II, and III.

In Method I, the fit parameters were estimated using the model-based error model with fixed ${b}_{D}$ and ${b}_{T}$ at the median of the 12 patients. These patient-specific fit parameters were then used to simulate the TACs of [^177^Lu]Lu-DOTA-TATE.

In Method II, the model-based error model was used with four different proportional parameters fixed to the median of 12 patients: ${b}_{D,Organ}$, ${b}_{D,Tumour}$, ${b}_{T,Organ}$ and ${b}_{T,Tumour}$. The 10 fit parameters were fitted individually, and the individual TACs of [^177^Lu]Lu-DOTA-TATE were simulated.

In Method III, to evaluate the [^177^Lu]Lu-DOTA-TATE prediction, the data-based error model was used, therapeutic proportional errors were fixed on the median over 12 patients, i.e. ${b}_{T,Organ}$ and ${b}_{T,Tumour}$, and diagnostic proportional parameters, ${b}_{D,Organ}$ and ${b}_{D,Tumour}$, were used as patient-specific parameter.

#### Evaluation of the prediction accuracy and precision

The prediction accuracy was assessed using the relative prediction error (RPE) for the TIAs of the kidneys, spleen, liver, and tumours. The TACs of organs and tumours were integrated from the start of the infusion to the end of the simulation (i.e., 40,000 min).

The predicted TIAs ($${TIA}_{pred})$$ were compared to the reference TIAs ($${TIA}_{ref}$$) according to

6 $$RPE=\frac{{TIA}_{pred}-{TIA}_{ref}}{{TIA}_{ref}}$$

The predictions were compared with the references for the Methods I, II and III described above.

Root mean square errors (RMSEs) of RPE

7 $$RMSE=\sqrt{mea{n}^{2}+{SD}^{2}}$$

and mean absolute percentage error (MAPE) over total number of TIAs, *M*, according to

8 $$MAPE=\frac{1}{M}\sum_{i}^{M}\frac{\left|{TIA}_{pred,i}-{TIA}_{ref,i}\right|}{{TIA}_{ref,i}}$$

were calculated for global comparison of the results.

## Results

### Estimation of the proportionality constant b in the error model

**Fig. 3:**
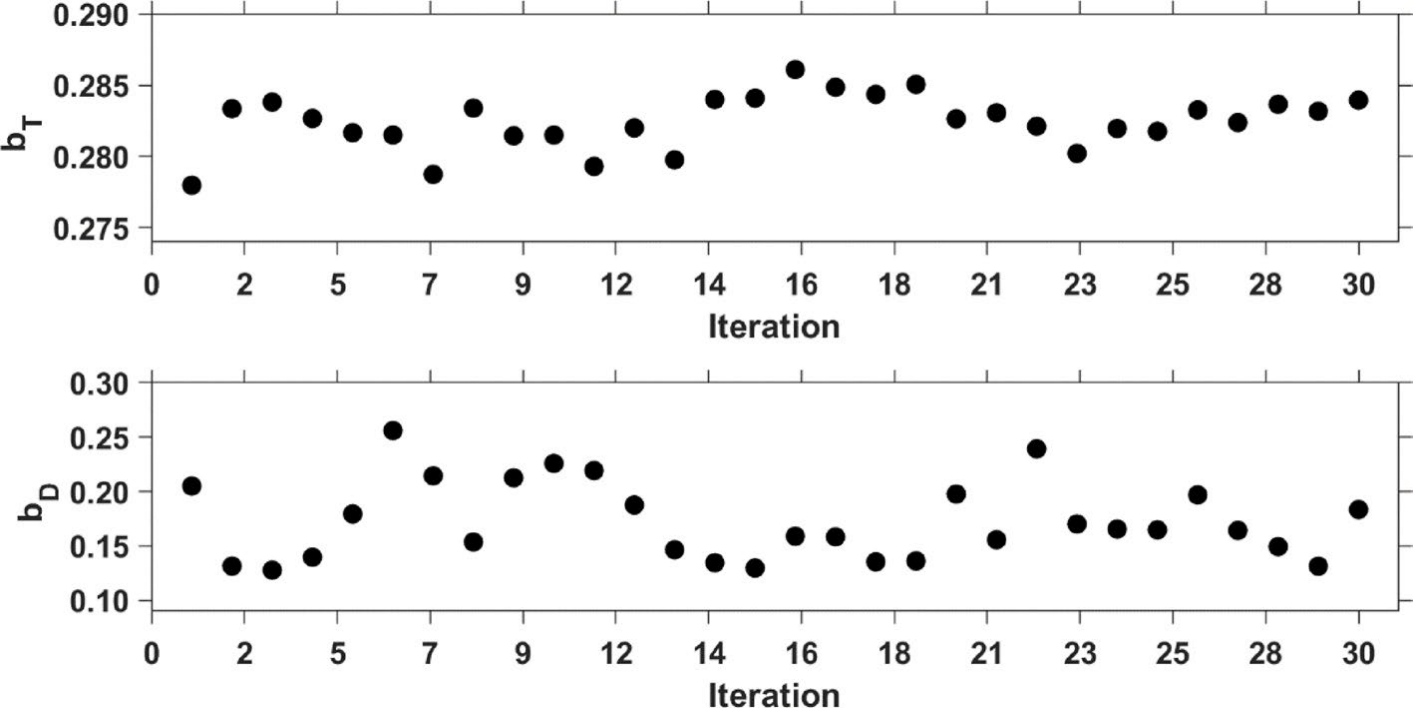
Iteration of proportionality constant *b* of the error model used to predict the time-integrated activity. Therapeutic proportional constant *b*_*T*_ and diagnostic proportional constant *b*_*D*_ are evaluated according to the scheme in Fig. 2. 28 iterations were performed (iterations were stopped when the fluctuation of *b*_*T*_ was < 0.005). *b*_*D*_, on the contrary, presents larger fluctuations

In Table 2, the proportional parameters obtained with the three previously described error models are summarised. The first column presents the ${b}_{D}$and ${b}_{T}$ parameters of Method I. After the first set of fits, the median values were 0.28 and 0.16 for ${b}_{T}$ and ${b}_{D}$, respectively. Figure 3 reports the proportional error factors after each iteration (according to the diagram in Fig. 2): both proportionality parameters fluctuate around the median, with ${b}_{D}$ showing a higher amplitude.

**Table 2:** Median [minimum, maximum] of constant *b* in the proportional error model (Eqs. 2 and 4) obtained for methods I–III. First, the model-based error model was used, and the proportionality constants *b* of diagnostic and therapeutic data were separately fitted. Second, the model-based error model was also used, but a further separation was done: organs and tumours were considered differently, and 4 b-parameters were fitted. Third, the data-based error model was used, and 4 b-parameters (with diagnostic and therapeutic data, organ and tumour separation) were fitted

	Model-based error model (2 bs) Method I		Model-based error model (4 bs) Method II	Data-based Error Model (4 bs) Method III
${{b}}_{{D}}$ (8 data)	0.16 [0.02, 0.51]	*b*_*D, Organ*_ (5 data)	0.28 [0.09, 0.56]	0.35 [0.13, 1.50]
		*b*_*D, Tumor*_ (3 data)	0.36 [0.13, 0.82]	0.27 [0.11, 1.39]
${{b}}_{{T}}$ (33 data)	0.28 [0.18, 0.63]	*b*_*T, Organ*_ (24 data)	0.31 [0.15, 0.83]	0.16 [0.14, 0.25]
		*b*_*T, Tumor*_ (9 data)	0.09 [0.01, 0.25]	0.39 [0.04, 0.80]

In Method II, it was hypothesized that the proportional error parameter, ${b}_{T}$ and ${b}_{D}$, differ between tumours and organs. The corresponding fit yielded different values for the proportional errors: according to Eq. 2, the median [maximum, minimum] values for b_T, Organ_, b_T, Tumour_ and b_D, Organ_, b_D, Tumour_ were determined to be 0.31, 0.09, 0.28, and 0.36 (Table 2).

In Method III, the median values for the data-based error model were 0.16, 0.39, 0.35, and 0.27 for *b*_*T, Organ*_, *b*_*T, Tumour*_, *b*_*D, Organ*_, and *b*_*D, Tumour*_, respectively (Table 2).

### Estimated parameters and predicted TACs

**Fig. 4:**
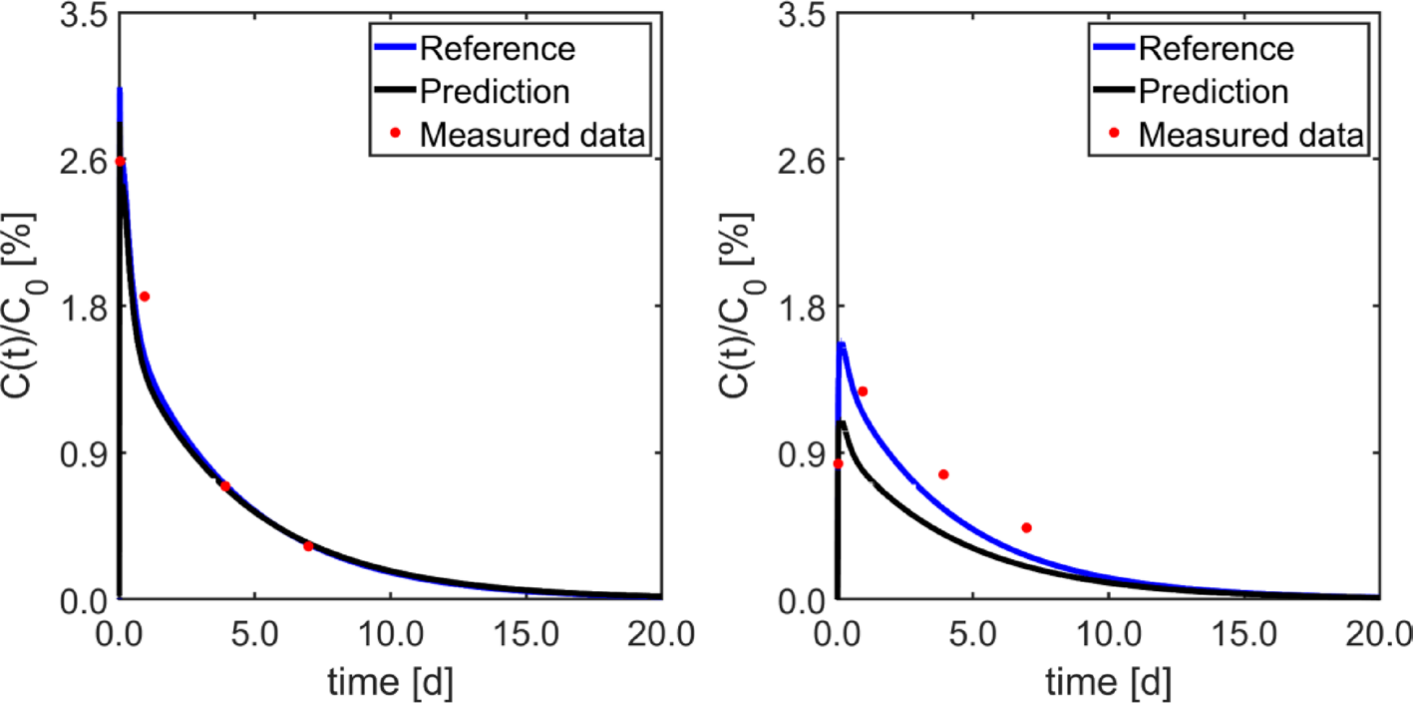
Comparison between the predicted and the reference time-activity curves for kidneys (left) and tumour (right) in a typical patient. Respective measured data, used as imputed data for the PBPK model, are also depicted

Table 3 reports the median [minimum, maximum] of the estimated parameters for the prediction of the TACs of [^177^Lu]Lu-DOTA-TATE using [^68^Ga]Ga-DOTA-TATE PET/CT and the Bayesian values. Figure 4 illustrates a typical example of predicted [^177^Lu]Lu-DOTA-TATE TACs of kidneys and tumours.

**Table 3:** Median [minimum, maximum] of fit parameters used for the TAC predictions. Two tumours were analysed separately, but the median [minimum, maximum] was calculated considering them together. Therefore, the receptor densities of tumour 1 and tumour 2 were grouped, as well as the blood flow in tumours 1 and 2

Parameter name	Model-based error model (2 bs) Method I	Model-based error model (4 bs) Method II	Data-based error model (4 bs) Method III
	Median [min, max]	Median [min, max]	Median [min, max]
Receptor density tumour 1 and 2 [nmol/l]	25 [5, 120]	28 [15, 59]	43[28, 73]
Receptor density kidneys [nmol/l]	6 [4, 8]	6 [5, 8]	9.9 [9.6, 10.1]
Receptor density liver [nmol/l]	2 [1, 4]	2 [1, 4]	3 [2, 5]
Receptor density muscles and fat [nmol/l]	0.2 [0.1, 1.5]	0.2 [0.1, 0.5]	0.3 [0.2, 0.4]
Receptor density spleen [nmol/l]	9 [4, 15]	9 [5, 13]	11 [8, 13]
Degradation rate [10^− 05^/s]	8 [7, 9]	2 [2, 4]	8.5 [8.4, 8.6]
Flow tumour 1 and 2 [1/s]	0.7 [0.05, 1.9]	0.2 [0.05, 0.7]	0.4 [0.1, 0.9]
Sort [10^− 03^/s]	4 [2, 5]	3 [2, 4]	2.8 [2.7, 3.0]

**Fig. 5:**
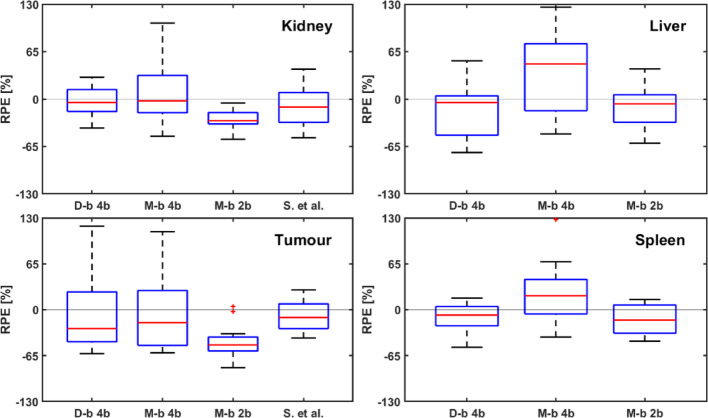
Relative predicted error for organs and tumours of data from prediction with data-based error model with 4 b-parameters (D-b 4b; Method III), with model-based error model with 4 b-parameters (M-b 4b; Method II), and with model-based error model with 2 b-parameters (M-b 2b; Method I). The RPEs for organs were calculated based on data from 12 patients, whereas the RPEs for tumours were based on 24 individual tumour lesions. Data from Siebinga et al. are also reported for comparison. RPEs for the kidneys in Siebinga et al. were calculated from 8 data, and the tumour from 9 data

Tables 4, 5, 6; Fig. 5 summarise the RPE of all patients. Using the model-based error model with two *b* parameters (*b*_*D*_ and *b*_*T*_), the mean and standard deviation RPE of TIA in Method I for tumours, kidney, liver, and spleen were (− 40 ± 35)%, (− 28 ± 14)%, (− 11 ± 29)% and (− 14 ± 21)%, respectively. Using the model-based error model with 4 *b* parameters, the median and standard deviation RPE in Method II showed a decrease, i.e., (− 5 ± 51)%, (8 ± 42)%, (36 ± 59)%, and (24 ± 45)%, for tumours, kidney, liver and spleen, respectively. Using the data-based error model and *b*_*D*_ as a patient-specific proportional error, the median RPEs in Method III were again smaller than in the previous estimations, i.e., (− 5 ± 51)%, (− 4 ± 22)%, (− 13 ± 40)%, and (− 10 ± 21)%, for tumours, kidney, liver, and spleen, respectively.

**Table 4:** Median [minimum, maximum], mean, standard deviation of relative prediction error (RPE) over time-integrated activity (TIA) using model-based error model with 2 *b*s of tumours and organs for the 12 patients (Method I). Root-mean-square errors (RMSEs) and mean absolute percentage error (MAPE) are also reported

	Tumours [%]	Kidney [%]	Liver [%]	Spleen [%]
Median [minimum, maximum]	− 50* [− 82, 75]*	− 30 [− 55, -5]	− 7 [− 61, 41]	− 15 [− 45, 14]
Mean	− 40*	− 28	− 11	− 14
Standard deviation	35*	14	29	21
RMSE	53*	31	31	25
MAPE	47*	28	23	21

*Tumour 1 and Tumour 2 were grouped

**Table 5:** Median [minimum, maximum], mean, standard deviation of relative prediction error (RPE) over time-integrated activity (TIA) model-based error model with 4 b-parameters (b_T, Organ_, b_T, Tumour_, b_D, Organ_, b_D, Tumour_), i.e., Method II. Root mean square errors (RMSEs) and mean absolute percentage errors (MAPEs) are also reported

	Tumours [%]	Kidney [%]	Liver [%]	Spleen [%]
Median [minimum, maximum]	− 17* [− 61, 111]*	− 3 [− 51, 104]	48 [− 48, 120]	20 [− 39, 128]
Mean	− 5*	8	36	24
Standard deviation	51*	42	59	45
RMSE	51*	43	69	51
MAPE	43*	29	56	37

* Tumour 1 and tumour 2 were grouped

**Table 6:** Median [minimum, maximum], mean, standard deviation of relative prediction error (RPE) over time-integrated activity (TIA) data-based error model with 4 b-parameters (b_T, Organ_, b_T, Tumour_, b_D, Organ_, b_D, Tumour_), i.e., Method III. Root mean square errors (RMSEs) and mean absolute percentage errors (MAPEs) are also reported

	Tumours [%]	Kidney [%]	Liver [%]	Spleen [%]
Median [minimum, maximum]	− 24* [− 62, 118]*	− 5 [− 40, 30]	-5 [− 73, 52]	-8 [−531, 16]
Mean	− 5*	-4	-13	-10
Standard deviation	51*	22	40	21
RMSE	52*	22	42	23
MAPE	43*	18	31	17

*Tumour 1 and tumour 2 were grouped

Tables 4, 5 and 6 report the MAPE value for TIA prediction using the three investigated methods. For tumours, kidneys, and spleen, Method III results in the smallest MAPE. However, for the liver, Method I showed superior accuracy, with a MAPE of 23% compared to 31% in Method III. Notably, high predictive accuracy for organ TIAs was accompanied by lower accuracy for tumour TIAs, and vice versa. In a subset of patients, kidney and tumour TIAs could be predicted with an RPE below 15%.

## Discussion

A PBPK model was developed and implemented in Simbiology/MATLAB, incorporating the injection of [^68^Ga]Ga-DOTA-TATE and the infusion of [^177^Lu]Lu-DOTA-TATE. The analysis of different error models and the comparison between the model-based and data-based error models revealed considerable differences regarding the accuracy and precision of the TIA predictions.

During the analyses, it was found that the constant error model parameter *a* = 0. The analyses showed that the measurement errors were adequately described by proportional error *b*, which can be obtained by solving the equation $\frac{{\partial OF}}{{\partial b}} = 0$. In Method I, the solution for the model-based error was estimated by Eq. 2 . By separating therapeutic and diagnostic data, two different proportional parameters were identified, namely ${b}_{D}=0.28$ and ${b}_{T}=0.16$. After 28 iterations, ${b}_{T}$ showed a constant trend, as is shown in Fig. 3. The parameter ${b}_{D}$, in contrast, exhibited larger fluctuations. This is likely because ${b}_{T}$ was derived from 33 time–activity data points (as shown in Table 2), whereas ${b}_{D}$ was obtained from only 8 time–activity data points. The fewer degree of freedom, therefore, led to greater fluctuations during the calculations.

Method II was implemented because the organ volumes are larger than those of tumours (0.01–0.06 L). Consequently, it was hypothesised that the proportional error constant *b* for tumours may differ from that of organs. Therefore, further data analysis yielded four distinct values for the proportional parameter *b*. According to Eq. 2, it was shown that median [min, max] of b_T, Organ_, b_T, Tumour_ and b_D, Organ_, b_D, Tumour_ are 0.31 [0.15, 0.83], 0.09 [0.01, 0.25], 0.28 [0.09, 0.56] and 0.36 [0.13, 0.82] (Table 2). It was observed that the magnitude of the fluctuations was in the order of the magnitude observed for b_D_. Based on our results, the fitting to a limited number of data points (3 for the calculation of *b*_*D, Tumour*_) can lead to increased inaccuracies in the obtained b values. Therefore, subsequent iterations were considered unnecessary, and the calculation process was stopped after the first iteration.

In Method III, the results for the data-based error model exhibited lower fluctuations among patients for the calculation of *b*_*T, Organ*_ (from Eq. 4), but remained equally variable for the other three parameters. This discrepancy might arise because 24 measured points were used for calculating *b*_*T, Organ*_. In contrast, only 9, 5, and 3 points were used for *b*_*T, Tumour*_, *b*_*D, Organ*_, and *b*_*D, Tumour*_, respectively. The median over 12 patients for the data-based error model for *b*_*T, Organ*_, *b*_*T, Tumour*_, *b*_*D, Organ*_, and *b*_*D, Tumour*_ were found to be 0.16, 0.39, 0.35 and 0.27, respectively (Table 2). Therapeutic proportional error results differed from the therapeutic proportional errors found in the case of the model-based error model. In general, the uncertainty of an estimated standard deviation is rather large even for a relatively high number of degrees-of-freedom. Therefore, one potential reason for the difference between values of *b* could be the result of random deviation.

The calculation of TACs showed differences in the prediction by using different methods. Method I provided the least accurate prediction, whereas Methods II and III yielded better results (Tables 4, 5, 6). Using Method III and fixing *b*_*D, Organ*_, and *b*_*D, Tumor*_ as patient-specific parameters, the prediction showed an even smaller RPE for organs. These improvements in prediction highlight the importance of accounting for individualised characteristics and physiological differences among patients when predicting TACs.

RMSEs provided quantitative measures of prediction accuracy. Lower RMSEs were observed for NETs, kidneys, and spleen when employing the data-based error model. This emphasises the superior performance of the last approach (Method III) in predicting TAC for these organs. Notably, the liver’s prediction accuracy remained consistent between the two error models (Method I and III), as indicated by comparable MAPE of RPE and RMSE values. This consistency may be attributed to the relatively stable physiological characteristics and metabolic processes of this organ, which are less susceptible to variations in model assumptions or input parameters.

Our predictions are equally good or better compared to other published results. Peterson et al. conducted a study on regression models using pretherapy [^68^Ga]Ga-DOTA-TATE-PET/CT uptake data and other baseline clinical factors/biomarkers to predict renal AD delivered by [^177^Lu]Lu-DOTA-TATE PRRT [42]. Univariate and bivariate models were tested and were compared with standard dosimetry. The MAPE obtained from the comparison between the univariate model and the calculated AD (18%) [42] differs minimally from the MAPE obtained in this analysis (17% for kidney).

Siebinga et al. demonstrated a significant difference in the population pharmacokinetics between [^177^Lu]Lu-DOTA-TATE and [^68^Ga]Ga-DOTA-TATE by predicting the pharmacokinetics of [^177^Lu]Lu-DOTA-TATE using [^68^Ga]Ga-DOTA-TATE imaging [21]. The study observed a range of RPE in AD to tumours from − 51 to 44%, and to the kidneys from − 58 to 82%. When comparing the findings presented here with those of Siebinga et al., the Root Mean Square Error (RMSE) for tumours suggests comparable prediction accuracy (RMSE = 49.1% versus RMSE = 51.6%). However, differences in tumour volumes analysed may help explain the observed small RMSE discrepancies. Specifically, Siebinga et al. analysed tumours with a median volume of 80.0 mL (range: 7.81–212 mL), which is notably larger than the tumours examined in the present study, where the median volume was 15.2 mL (range: 0.01–56.05 mL). The relatively smaller tumour volumes in our study may pose greater challenges in accurate quantification, potentially contributing to higher RMSE values.

Furthermore, Siebinga et al. integrated the TAC up to the final measured time point (3–5 days), excluding later time points. This likely resulted in a reduced RMSE compared to the present study, where the TAC integration extended up to 40,000 min (27.8 days) after injection. While the accuracy of the tumour TAC results in this study may be affected by the extended time integration, the substantial differences in tumour volume raise questions about the comparability of these results across studies.

For kidneys, in contrast, the RPEs obtained in Method III for the data-based error model resulted in smaller values than those published by Siebinga et al., indicating that the model could provide better predictions for kidneys. By also analysing the RMSE of the AD, where the integration was performed up to the end of the TAC, Siebinge et al. reported an RMSE of 46%, whereas the results of this study showed an RMSE of 22%. This suggests that the kidney model reported in this PBPK model is more accurate in predicting the therapeutic TACs.

The modelling approach adopted in this study assumes that the biological system (i.e., the patient) remains unchanged between the pre-therapeutic PET/CT scan and the therapeutic administration of [^177^Lu]Lu-DOTA-TATE. This assumption introduces limitations, as the functionality of tumour tissue and healthy organs may vary over time, potentially influencing the fitted pharmacokinetic parameters. In particular, all patients received treatment with long-acting somatostatin analogues (LAR), which was discontinued before the [^177^Lu]Lu-DOTA-TATE therapy but not before the PET/CT acquisition. This could create a pharmacological discrepancy between the two time points that may affect uptake and distribution, thereby challenging the assumption of physiological equivalence [43–46].

Another limitation arises from the assumption of inter-patient physiological similarity. In Methods I and II, the parameter *b* was averaged across all patients, assuming a shared underlying pharmacokinetic behaviour. Only in Method III was an individualised value of *b* applied for the diagnostic measurements; however, a population-based median value of b was still employed for the therapeutic measurements.

Additionally, slight differences were observed between organ and tumour volumes estimated from PET/CT and post-therapy imaging, with an average variation of approximately 1%. This difference is primarily attributed to variability in volume delineation and the different imaging modalities used, assuming anatomical volumes remain stable between the two time points; a reasonable hypothesis considering the typically slow progression of NETs. Significantly, no correlation was found between prediction accuracy and volume variation.

Overall, the findings suggest that the PBPK model, integrated with a data-based proportional error model, enhances the precision of TAC predictions for NETs and selected organs following [^177^Lu]Lu-DOTA-TATE therapy. By leveraging single-time-point PET/CT imaging with [^68^Ga]Ga-DOTA-TATE, clinicians may thus obtain reliable estimates of TACs and consequently ADs, facilitating personalised treatment planning and optimising therapeutic outcomes. Further refinement of the PBPK model and validation studies with larger patient cohorts may contribute to its broader clinical utility and effectiveness in precision oncology.

## Conclusion

The integration of the PBPK model with a data-based proportional error model represents a significant improvement in predicting TACs for estimating tumour and organ ADs following [^177^Lu]Lu-DOTA-TATE therapy, using single-time-point PET/CT imaging with [^68^Ga]Ga-DOTA-TATE. Particularly in the context of NETs and targeted organs, a prediction, RPE (mean und SD), of the TACs can be obtained (− 5 ± 51)% for tumours and (− 4 ± 22)% for kidneys. Through single-time-point PET/CT imaging with [^68^Ga]Ga-DOTA-TATE, it is possible to enable personalised treatment strategies tailored to individual patients. This approach not only facilitates optimised therapeutic outcomes but also underscores the importance of precision oncology in guiding clinical decisions.

## Supplementary Information

Below is the link to the electronic supplementary material.

Supplementary Material 1

## Data Availability

Data will be made available on reasonable request.

## Abbreviations

AD: Absorbed dose
GI: Gastrointestinal tract
TuL: Liver tumours
MAPE: Mean absolute percentage error
NET: Neuroendocrine tumour
PRRT: Peptide receptor radionuclide therapy
PET/CT: Positron emission tomography/computed tomography
TIA${}_{pred}$: Predicted time-integrated activity
RM: Red bone marrow
TIA${}_{ref}$: Reference time-integrated activity
RPE: Relative prediction error
RoB: Rest of the body
RMSE: Root mean square error
SPECT/CT: Single photon emission computed tomography/computed tomography
SSA: Somatostatin analogue
SSTR2: Somatostatin-receptor 2
SUV: Standardised uptake value
TAC: Time-activity curve
TIA: Time-integrated activity
Tu1: Tumour 1
Tu2: Tumour 2
TuRofB: Tumour lesions in the rest of the body
