# Supplementary material for: [177Lu]Lu-DOTA-TATE tumour and organ time-activity curves: prediction from a single-time-point [68Ga]Ga-DOTA-TATE PET/CT measurement
Source: EJNMMI Phys. 2026 Jan 16;13:9. doi: 10.1186/s40658-025-00826-4 (PMC12847613; doi:10.1186/s40658-025-00826-4)
Supplement: Supplementary file 1 — Supplementary Material 1 [file 40658_2025_826_MOESM1_ESM.docx]

Supplement

[^177^Lu]Lu-DOTA-TATE tumour and organ time-activity curves: Prediction from a single-time-point [^68^Ga]Ga-DOTA-TATE PET/CT measurement

Valentina Vasić^1,2^, Johan Gustafsson^3^, Elham Yousefzadeh-Nowshahr^1,2^, Ambros J. Beer^1^, Katarina Sjögreen Gleisner^3,*^, Gerhard Glatting^1,2,*^

###### ^1^Department of Nuclear Medicine, Ulm University, Ulm, Germany

###### ^2^Medical Radiation Physics, Department of Nuclear Medicine, Ulm University, Ulm, Germany

###### ^3^Medical Radiation Physics, Lund University, Lund, Sweden

*^*^Shared senior authorship*

In this Supplement, information about the structure of the PBPK model (section S1) and the simulation and fitting process with the PBPK model codes implemented in SimBiology is detailed (sections S2, S3, and S4). Section S5 of the Supplement reports detailed results in Tables A1a, A1b, and A1c on the relative prediction error (RPE) of the time-integrated activity (TIA) for each patient.

- 1. **
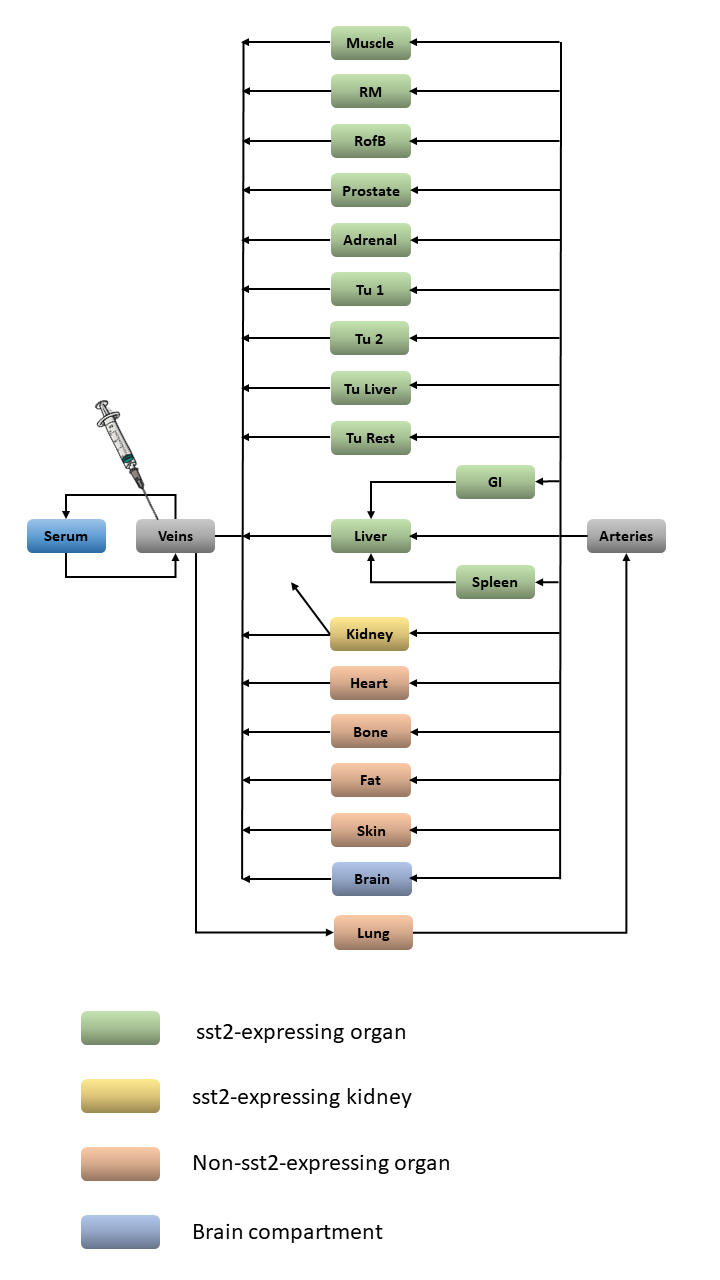
PBPK model**

**FIGURE S1**: PBPK model for DOTATATE. The model describes the pharmacokinetics of [^177^Lu]Lu-DOTATATE and [^68^Ga]Ga-DOTATATE implemented for a patient with NET and treated with Lutathera or similar radiopharmaceuticals. First, [^68^Ga]Ga-DOTATATE and then [^177^Lu]Lu-DOTATATE are distributed in the blood circulation to sst2-expressing organs and non-sst2-expressing organs. RM represents red bone marrow, while RofB refers to the remaining parts of the body. Tu 1 and Tu 2 denote tumour 1 and tumour 2, respectively. Tu Liver and Tu Rest represent liver tumours and tumours located outside the previously mentioned areas. GI stands for the gastrointestinal tract.

- 1. **Simulations with the PBPK model**

The simulation duration was fixed to 27.8 d (from 0 to 40,000 min). As Vasić et al. ("A PBPK model for PRRT with [^177^Lu]Lu-DOTA-TATE: Comparison of model implementations in SAAM II and MATLAB/SimBiology." Phys Med 2024; **119**: 103299) demonstrated, over 99% of the radiopharmaceutical is eliminated or decayed within this period.

Because [^68^Ga]Ga- and [^177^Lu]Lu-DOTA-TATE were implemented together in the same model, the parameters half-life, decay constant rate ($\lambda_{\Phi}$) and uptake of unspecific binding in the kidney ($k_{k,unsp.}$) were implemented like a switcher, i.e. from time 0 min to the time of infusion (7 days after) the parameter have value related to ^68^Ga ($\lambda_{\Phi}$ = 1.03*10^-02^$\frac{1}{\min}$ and $k_{k,unsp.}$ = 0.03 $\frac{1}{\min}$), on the other side, for times from the infusion up to end (27.8 days), the parameter value were related to ^177^Lu ($\lambda_{\Phi}$ = 7.15322*10^-05^$\frac{1}{\min}$ and $k_{k,unsp.}$ = 0.015 $\frac{1}{\min}$).

For simulations, the "sundials" ([31](#_ENREF_31)) solver options were selected, with absolute and relative tolerances set at 10^-12^.

- 1. **Fitting with the PBPK model**

To predict the TACs, a fitting approach based on time-activity PET/CT measured data per patient was done. The fitting procedure used Bayesian information ([20](#_ENREF_20)) and a proportional error model in the objective function. The stopping criteria were specified as follows:

- a lower threshold on the size of a step, $TolX = {10}^{-6}$
- a lower limit on the change in the value of the objective function (OF) during a step, $TolFun={10}^{-6}$
- an upper bound on the number of solver iterations,  $MaxIter ={10}^{6}$
- an upper bound on the number of function evaluations, ${MaxFunEvals=10}^{6}$.

$OF= \sum_{j=1}^{J} \sum_{i=1}^{N_{j}} \left\{ \ln\left( 2\pi\sigma_{i,j}^{2} \right)+\frac{{(y_{i,j}-f(t_{i,j},\boldsymbol{p}))}^{2}}{\sigma_{i,j}^{2}} \right\}+\sum_{k=1}^{K} \left\{ \ln\left( 2\pi\omega_{k}^{2} \right)+\frac{{(p_{k}-\bar{p}\boldsymbol{)}}^{2}}{\omega_{k}^{2}} \right\}$ (S1)

where $N_{j}$ is the total number of measured data for patient *j* (with J the total number of patient, i.e. 12), $y_{i}$ are the measured data and ${f(t}_{i}|\vec{p})$ are the model data estimations; $t_{i}$ are the time points and ***p***is the model parameter vector. *K* is the total number of Bayes parameters $p_{k}$, with population mean$\bar{p}. \sigma_{i,j}$ is the uncertainty of the measurement and $\omega_{k}$ the standard deviation over the population of $p_{k}$.

1. **Akaike information criterion**

The corrected Akaike Information Criterion (AICc) is calculated according to (Glatting et al. "Choosing the optimal fit function: Comparison of the Akaike information criterion and the F-test" Med Phys. 2007; 34(11): 4285-4292)

$AICc=AIC+2*\frac{K(K+1)}{N-K-1}$ , (S2)

Where $AIC=2*(K-LogLikelihood)$, $K$ the **number** of free parameters of the model and $N=n+B$ the sum of the number of measured points and the number of Bayes parameters used.

1. **Detailed results for patients**

**TABLE A1a**: Relative prediction error (RPE) of time-integrated activity (TIA) using model-based error model with 2 *b*s (Method I) of tumours and organs for the 12 patients.

| Patient | Tumour 1 [%] | Tumour 2 [%] | Kidney [%] | Liver [%] | Spleen [%] |
| --- | --- | --- | --- | --- | --- |
| 1 | -49 | -50 | -32 | 5 | -15 |
| 2 | -67 | -59 | -36 | -8 | -33 |
| 3 | -50 | -37 | -17 | -38 | 6 |
| 4 | -63 | -62 | -7 | -12 | -34 |
| 5 | -34 | -3 | -20 | -61 | -15 |
| 6 | -48 | -36 | -27 | -26 | -8 |
| 7 | -51 | -69 | -29 | 17 | 7 |
| 8 | -3 | 75 | -32 | -54 | -38 |
| 9 | -82 | -79 | -55 | -2 | -45 |
| 10 | -44 | -40 | -5 | -5 | 12 |
| 11 | 4 | 8 | -40 | 41 | 14 |
| 12 | -54 | -62 | -31 | 7 | -21 |
| Median  [minimum, maximum] | -50*  [-82, 75]* |  | -30  [-55, -5] | -7  [-61, 41] | -15  [-45, 14] |
| Mean | -40* |  | -28 | -11 | -14 |
| Standard Deviation | 35* |  | 14 | 29 | 21 |
| RMSE [%] | 53* |  | 31 | 31 | 25 |
| MAPE [%] | 47* |  | 28 | 23 | 21 |

*** Tumour 1 and Tumour 2 were grouped.**

**TABLE A1b.** Relative prediction error (RPE) of time-integrated activity (TIA) using model-based error model with 4 *b*s (b_T,Organ_, b_T,Tumour_, b_D,Organ_, b_D,Tumour_) (Method II).

| Patient | Tumour 1 [%] | Tumour 2 [%] | Kidney [%] | Liver [%] | Spleen [%] |
| --- | --- | --- | --- | --- | --- |
| 1 | -21 | -18 | -4 | 60 | 17 |
| 2 | -57 | -46 | -29 | 3 | -28 |
| 3 | -47 | -46 | -51 | -17 | 11 |
| 4 | -54 | -54 | -3 | -15 | -39 |
| 5 | -15 | 25 | 49 | -48 | 22 |
| 6 | 16 | 30 | 52 | 126 | 128 |
| 7 | 38 | 16 | 6 | 72 | 49 |
| 8 | 52 | 32 | -2 | -45 | -12 |
| 9 | -61 | -57 | 15 | 115 | 35 |
| 10 | 9 | 13 | 104 | 47 | 68 |
| 11 | 111 | 110 | -24 | 79 | 36 |
| 12 | -41 | -51 | -13 | 49 | -0.2 |
| Median  [minimum, maximum] | -17*  [-61, 111]* |  | -3  [-51, 104] | 48  [-48, 120] | 20  [-39, 128] |
| Mean | -5 |  | 8 | 36 | 24 |
| Standard Deviation | 51 |  | 42 | 59 | 45 |
| RMSE [%] | 51 |  | 43 | 69 | 51 |
| MAPE [%] | 43 |  | 29 | 56 | 37 |

*** Tumour 1 and Tumour 2 were grouped.**

**TABLE A1c.** Relative prediction error (RPE) of time-integrated activity (TIA) using a data-based error model with 4 *b*s (b_T,Organ_, b_T,Tumour_, b_D,Organ_, b_D,Tumour_) (Method III).

| Patient | Tumour 1 [%] | Tumour 2 [%] | Kidney [%] | Liver [%] | Spleen [%] |
| --- | --- | --- | --- | --- | --- |
| 1 | -28 | -22 | -2 | 50 | 5 |
| 2 | -55 | -30 | -19 | 4 | -32 |
| 3 | -29 | -19 | -3 | -56 | 2 |
| 4 | -49 | -49 | 30 | -26 | -21 |
| 5 | -26 | 4 | 16 | -73 | -24 |
| 6 | 5 | 35 | 14 | -3 | 16 |
| 7 | 47 | 39 | -11 | 1 | 4 |
| 8 | 118 | 112 | 12 | -50 | 16 |
| 9 | -62 | -59 | -37 | -6 | -53 |
| 10 | -42 | -39 | -7 | -49 | -12 |
| 11 | 45 | 55 | -40 | 5 | -17 |
| 12 | -23 | -42 | 25 | 52 | -3 |
| Median  [minimum, maximum] | -24*  [-62, 118]* |  | -5  [-40, 30] | -5  [-73, 52] | -8  [-53, 16] |
| Mean | -5 |  | -4 | -13 | -10 |
| Standard Deviation | 51 |  | 22 | 40 | 21 |
| RMSE [%] | 52 |  | 22 | 42 | 23 |
| MAPE [%] | 43 |  | 18 | 31 | 17 |

*** Tumour 1 and Tumour 2 were grouped.**
